# Zinner’s syndrome: report of two cases and review of the literature

**DOI:** 10.1186/s12610-016-0037-4

**Published:** 2016-09-20

**Authors:** Amine Slaoui, Souhail Regragui, Abdelouahad Lasri, Tarik Karmouni, Khalid El Khader, Abdellatif Koutani, Ahmed Ibn Attya

**Affiliations:** Urology B Ibn Sina Hospital, Mohammed V University, Rabat, Morocco

**Keywords:** Zinner’s syndrome, Seminal vesicle cysts, Ipsilateral renal agenesis

## Abstract

**Background:**

Congenital malformations of the seminal vesicle are uncommon, and most of them are cystic malformations. If an insult occurs between the 4th and the 13 h gestational week, the embryogenesis of the kidney, ureter, seminal vesicle, and vas deferens could be altered. Cysts of the seminal vesicle may appear with a mass effect, dysuria, epididymitis, or obstruction of the gastrointestinal and genitourinary tracts. Approximately two thirds of them are associated with ipsilateral renal agenesis, because both the ureteral buds and seminal vesicles originate from the mesonephric (Wolffian) duct. They were first described by Zinner in 1914, and 200 cases of seminal vesicle cysts associated with ipsilateral renal agenesis have been reported in the literature. Most patients with this anomaly are asymptomatic until the third or fourth decade of life. Some cases have nonspecific symptoms such as prostatism, urinary urgency, dysuria, painful ejaculation, and perineal discomfort. Transrectal ultrasonography provides good visualization of the pelvic structures and allows guidance for aspiration of the cysts.

**Case presentation:**

We present two cases of seminal vesicle cyst. The first patient had dysuria, increased frequency of urination, and haematuria. He was operated and benefited from a removal of the cyst with right ureterectomy and left ureteral reimplantation. The second patient had disorder of the digestive transit and he benefited from a laparoscopic removal of the cyst.

**Conclusions:**

Seminal vesicle cysts combined with ipsilateral renal agenesis are rare urological anomalies. Usual symptoms that are caused by the seminal vesicle cysts are bladder irritation and obstruction as well as pain in the perineum and scrotum. Epididymitis is frequently found. Treatment consists to removing the seminal vesicle cyst.

## Background

Zinner’s syndrome is a congenital malformation of the seminal vesicle and ipsilateral upper urinary tract that was first discovered in 1914 [1]. This includes the seminal vesicle cyst, ejaculatory duct obstruction, and ipsilateral renal agenesia. 200 cases of seminal vesicle cysts associated with ipsilateral renal agenesis have been reported in the literature [2]. Between 2000 and 2015, the Urology Department B of the Ibn Sina Hospital reported that there were two patients who had the Zinner’s syndrome.

## Case report 1

A 39- year-old father of five children was admitted in our department with intermittent and capricious hematuria lasting for 10 years. Its history was marked by the existence of several episodes of urinary infection during childhood and adolescence. Since about 1 year, the patient had a terminal hematuria and pyuria associated with right lumbar pain radiating to the external genitalia.

The examination revealed a patient in good general condition, afebrile. The abdomen was soft with no palpable mass. Digital rectal examination was a bulging mass in the rectum. Laboratory tests including urine culture showed no abnormality.

Ultrasonography revealed a hypoechoic formation accompanied by a posterior enhancement evoking an impure fluid collection. Intravenous urography (IVU) showed a normal left kidney but did not allow to study the right kidney (silent kidney?). The voiding images showed bilateral Vesicoureteral Reflux (VUR) with opacification of a blind right ureter. Computed tomography (CT) showed a cyst behind the bladder and right renal agenesis.

Indeed, voiding IVU image allow the conclusion to an intra- bladder anastomosis of the right ureter (opacification of the right ureter without opacification of the cyst) and cystoscopy was normal outside the ureteral meatus in gaping hole golf.

The indication for ureteral reimplantation being retained, the patient was operated. By pubic incision above the umbilicus straddling he underwent excision of the cyst and seminal vesicle with ureterectomy straight right and left ureteral reimplantation Cohen. The postoperative course was uneventful. With over 10 years of follow the patient is healthy.

## Case report 2

**Fig. 1:**
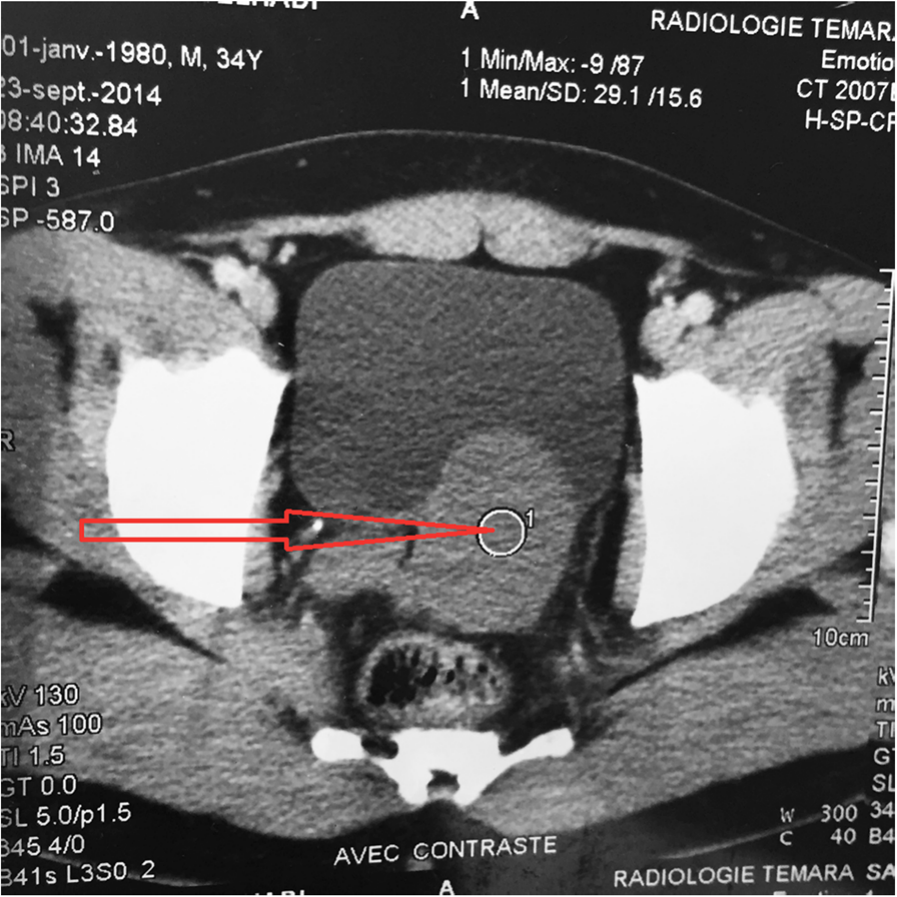
CT image showing a homogeneous liquid mass to polylobed contours not taking contrast retrovesical

**Fig. 2:**
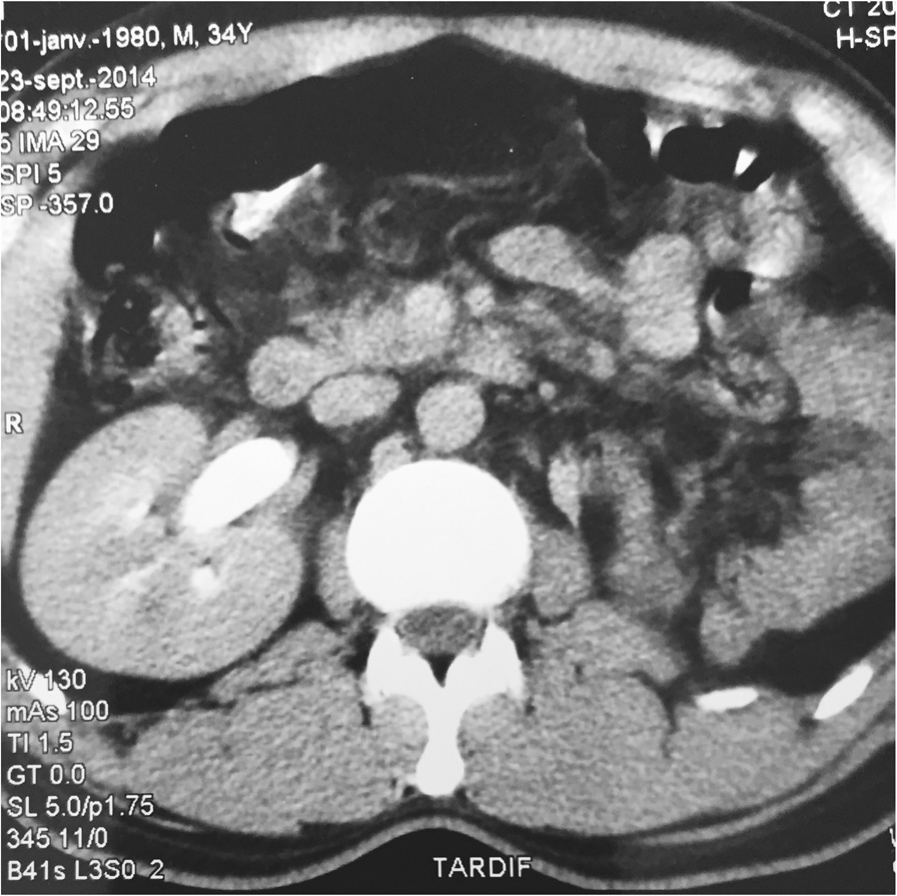
CT image showing a single right kidney

**Fig. 3:**
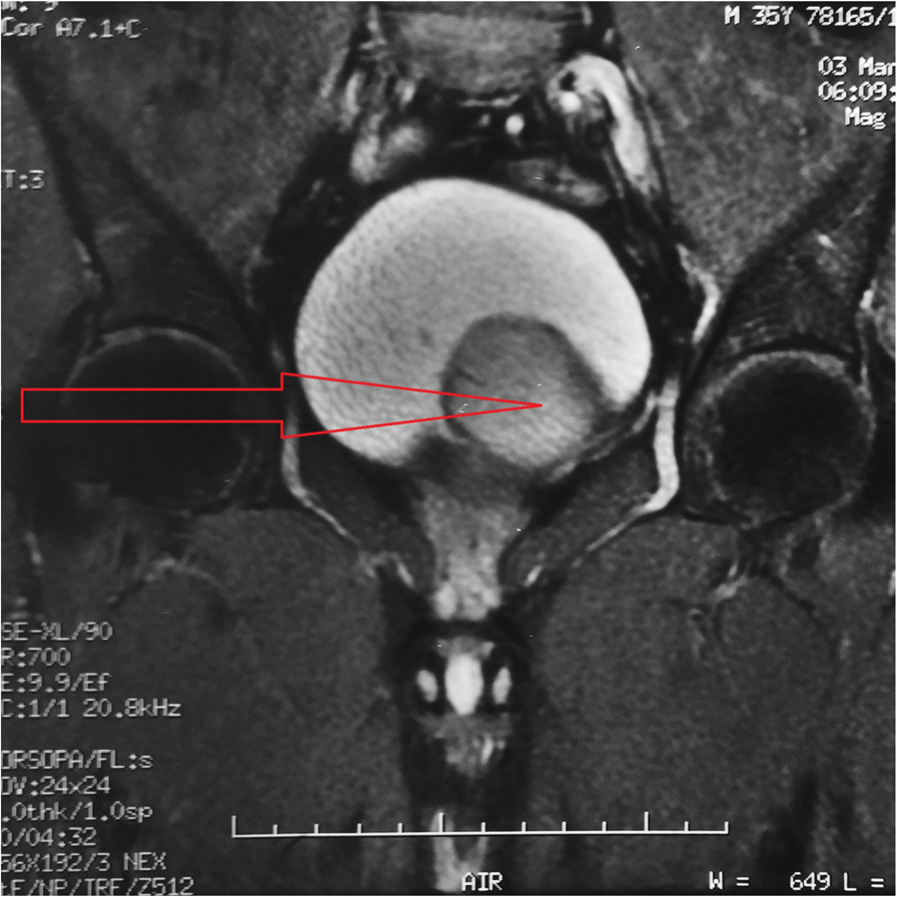
Pelvic MRI showing retrovesical cyst, limited training well lateralized left coarsely rounded (56 × 54 × 44) with thin wall

**Fig. 4:**
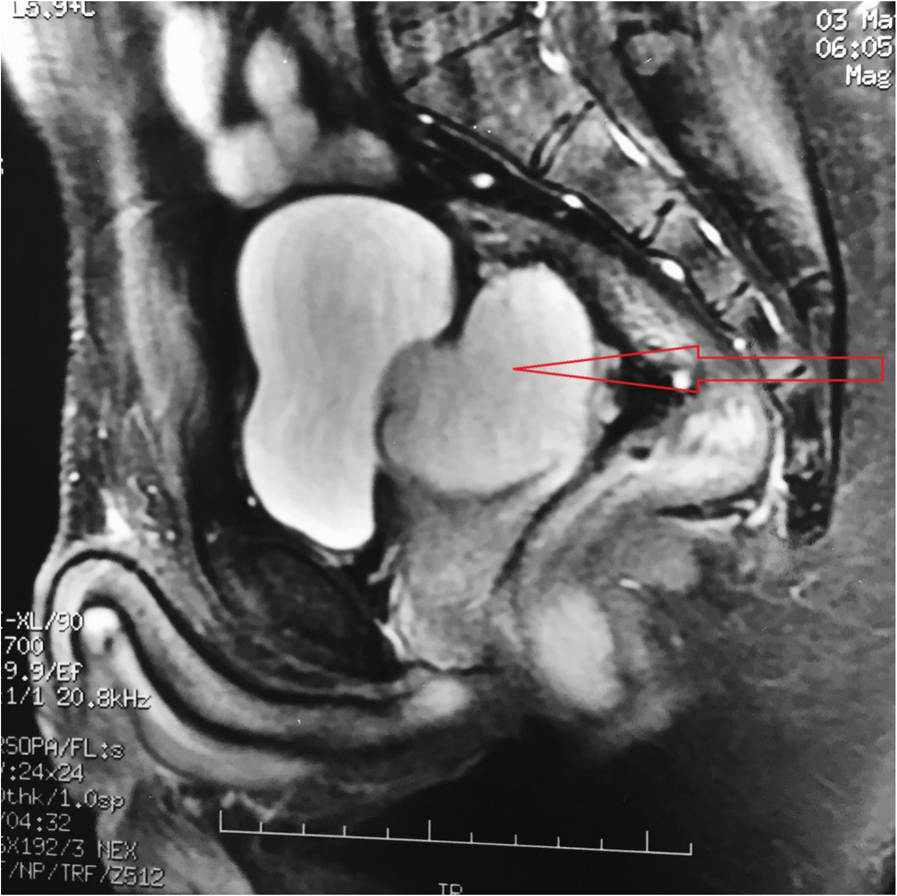
Pelvic MRI showing the cyst (Sagittal section)

**Fig. 5:**
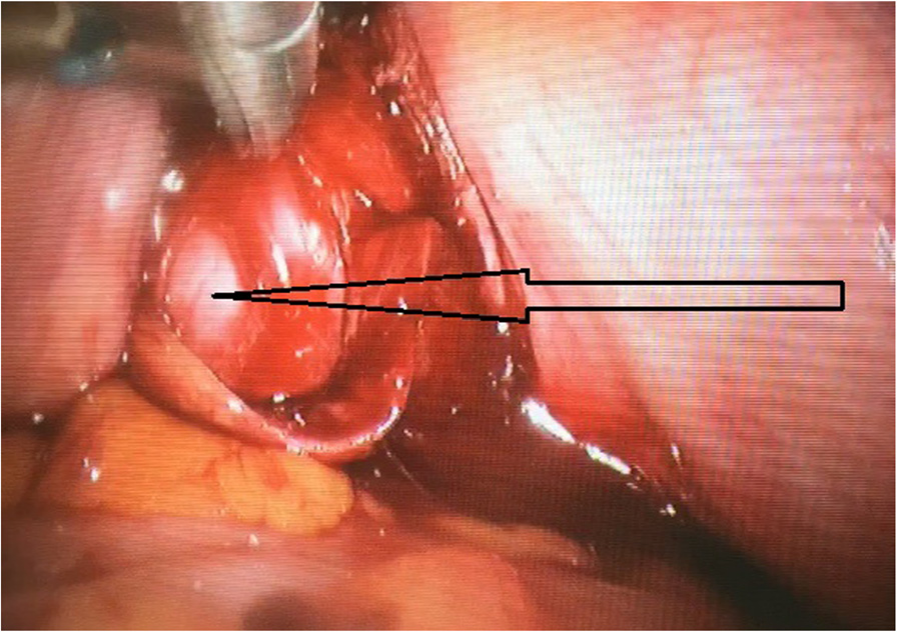
Image intraoperative (laparoscopic): retrovesical cyst

A 35 year old patient hospitalized for subocclusion underwent ultrasound showing a retrovesical liquid mass. Biological assessments are without fault. CT shows a homogeneous liquid mass with polylobed contours not taking contrast retrovesical seat (Fig. 1), molding the posterior surface of the bladder, measuring 80 × 60 mm, and associated with a single right kidney (Fig. 2). The radiological assessment was completed by pelvic MRI showing a left lateralized retrovesical coarsely rounded (56 × 54 × 44) with thin wall. It delivers surrounding structures. The radiological assessment was completed by pelvic MRI (Figs. 3 and 4). There is no lymphadenopathy iliac or pelvic effusion (Fig. 5). He benefited from a laparoscopic removal of the cyst. The postoperative course was uneventful.

## Discussion

During the period of utmost sexual or reproductive activity, cysts of the seminal vesicles start to become more apparent, especially between the second to third decades [3]. The seminal vesicle cysts that are less than 5 cm stay asymptomatic and are later discovered a palpable abdominal mass or on digital rectal examination as a palpable fluctuant mass arising from the superior aspect of the prostate gland. Bladder irritation and obstruction could also be related to symptoms of cysts [4, 5]. The most common and frequent symptoms of cysts are abdominal, perineal, and pelvic pain [6–8]. Other pains could be ejaculatory pain, dysuria, hematuria, urinary tract infections, and symptoms of epididymitis and prostatitis. Some of the other symptoms that were reported, but remaining rarely known, are infertility, hemospermia, and enuresis [5]. Cysts that exceed 12 cm are known to be as the “giant” cysts. These could be known through symptoms of bladder and colonic obstruction because of their enormous effect [4]. Few of the prepubertal young men that were examined for epididymitis and chronic urinary tract infections were found with seminal vesicle cysts.

Seminal vesicle cysts may be congenital or acquired [9]. Those that are present since birth, usually develop and become symptomatic during the adulthood. A cyst is created or formed when secretions in the gland owing to insufficient drainage which is lined or related to atresia, which later causes distention of the seminal vesicles [6]. The cysts that were acquired are often bilateral and are discovered after a history of chronic prostatitis or even a prostate surgery [3]. The relation between seminal vesicle cysts and ipsilateral renal agenesis can be explained by their common embryological origin. The ureteral bud originates from the dorsal aspect of the distal mesonephric duct and extends in a dorsocranial fashion to meet and induce differentiation of the metanephric blastema, which will form the adult definitive kidney. The mesonephric duct differentiates into the appendix of the epididymis, paradidymis, epididymis, vas deferens, ejaculatory duct, seminal vesicle, and hemitrigone [10, 11].

The kidney’s development all relies on the stimulation of the ureteric bud and mesonephric duct. The failure of the ipsilateral kidney, ureter, hemitrigone, and seminal vesicle are usually derived from the malfunction of the mesonephric duct. This will lead to ipsiltaeral renal agenesis or dyplasia. If the ureteral bud arises in a more cephalic position off the mesonephric duct, delayed absorption of the caudal mesonephric duct will result in the distal ureteral bud emptying into mesonephric duct derivatives, including the vas deferens, seminal vesicle, ejaculatory duct, or into the bladder neck and urethra [12]. Ectopic ureters entering seminal vesicle cysts associated with ipsilateral renal agenesis are uncommon; however, they have been reported and may be complicated by reflux and obstruction [7]. All this explains that the seminal vesicle cyst associated with agenesis or dysplasia of the ipsilateral kidney is rare. Their frequency is estimated, according to a Chinese study of 280,000 children at 0.00035 % [13].

For the evaluation and discrimination of pelvic cystic masses, there were many imaging techniques are used. Excretory urography can show ipsilateral renal dysgenesis and an abnormal appearance of the collecting system [9]; however an extrinsic smooth-walled filling defect in the bladder that is suggestive of a seminal vesicle cyst may not be visualized. The advanced technology known as the sonographic can determine cystic nature of the pelvic masses, the size and location, and define intraprostatic anatomy [9, 14, 15]. These latest discoveries or findings are an anechoic pelvic mass with a thick and irregular and occasional wall. Sometimes the mass may also consist of internal debris related to an early infection [9]. Reported findings with vasovesiculography include dilatation, mass effect with deformity of the seminal vesicle, ejaculatory duct stenosis, and reflux of contrast material in an ipsilateral ectopic ureter.

CT can accurately show renal anomalies and define pelvic anatomy. Reported findings have been variable ranging from a cystic pelvic mass with a thick irregular wall to a solid mass and apparent enlargement of the ipsilateral seminal vesicle. Other reported findings include a well-defined low-attenuation retrovesicular mass arising from the seminal vesicle, cephalic to the prostate gland, with associated renal anomalies [9].

The multiplanar ability of MR imaging to define abdominal and pelvic anatomy and to differentiate cystic malformations of the pelvis make it the ideal imaging study, allowing prompt diagnosis. In our study, the two patients that had symptoms of dysuria and irregular perineal pain were because of the late or missed diagnosis that was supposed to be done earlier. The usual appearance of a seminal vesicle cyst is that of cyst located elsewhere in the body, showing low T1-weighted and high T2-weighted signal intensity. However, seminal vesicle cysts may show increased T1-weighted and T2-weighted signal intensity, thought to reflect increased concentration of proteinaceous material or hemorrhage [16]. Some undergo surgery for these symptoms but it all depends in the size of the cyst. MR imaging are used to determine the approximate location where the cyst is located before any excision is done.

Only the symptomatic forms of treatment are justifiable. This treatment is surgical: excision of the cyst. This surgical approach may be made by trans-vesical way, extra bladder or laparoscopically [11, 17]. According to Williams, the way transcoccyx should be a last resort because exposure to the risk of rectal injury and impotence. Using the transperineal route is a fascinating way to cure the cyst, but it does not allow treatment of the associated anomalies in the same time [11, 18]. An alternative to other surgical procedures or approaches can be done using laparoscopy. But this technique has a limit when some of the surgeries require a ureteral reimplantation. Other approaches have also been proposed: transrectal. In case of recurrence after aspiration, some authors recommend to redraw the cyst and then to inject a sclerosing agent [19].

## Conclusion

Seminal vesicle cysts combined with ipsilateral renal agenesis are a rare urological anomaly. These usually occur in males between the 2^nd^ to 4^th^ decades of their life. The usual symptoms that are caused by the seminal vesicle cysts are bladder irritation and obstruction as well as pain in the perineum and scrotum. Epididymitis is frequently found. The diagnostic work-up consists of a digital rectal examination, transrectal and abdominal ultrasonography, CT scan and a cystoscopy. For the removal of the seminal vesicle cyst there are techniques that are used nowadays due to advanced technologies such as open surgery and transurethral deroofing of the cyst.

## Abbreviations

CT: Computed tomography
IVU: Intravenous urography
MRI: Magnetic resonance imaging
VUR: Vesicoureteral Reflux

## References

[1] Zinner A (1914). Ein fall von intravesikaler samenblasenzyste. Weien Med Wschr.

[2] Pereira BJ, Sousa L, Azinhais P, Conceição P, Borges R, Leão R, Brandão A, Temido P, Retroz E, Sobral F (2009). Zinner’s syndrome: an up-to-date review of the literature based on a clinical case. Andrologia.

[3] Rappe BJM, Meuleman EJH, Debruyne FMJ (1993). Seminal vesicle cyst with ipsilateral renal agenesis. Urol Int.

[4] Heaney JA, Pfister RC, Meares EM (1987). Giant cyst of the seminal vesicle with renal agenesis. AJR Am J Roentgenol.

[5] Kenney PJ, Leeson MD (1983). Congenital anomalies of the seminal vesicles: spectrum of computed tomographic findings. Radiology.

[6] Beeby DI (1974). Seminal vesicle cyst associated with ipsilateral renal agenesis: case report and review of literature. J Urol.

[7] Levisay GL, Holder J, Weigel JW (1975). Ureteral ectopia associated with seminal vesicle cyst and ipsilateral renal agenesis. Radiology.

[8] Okada Y, Tanaka H, Takeuchi H, Yoshida O (1992). Papillary adenocarcinoma in a seminal vesicle cyst associated with ipsilateral renal agenesis: a case report. J Urol.

[9] King BF, Hattery RR, Lieber MM, Berquist TH, Williamson B, Hartman GW (1991). Congenital cystic disease of the seminal vesicle. Radiology.

[10] Moore KL (1997). The developing human.

[11] Williams RD, Sandlow JI, Walsh PC, Retik AB, Vaughan ED, Wein AJ (1998). Surgery of the seminal vesicles. Campbell’s urology.

[12] Shariat SF, Naderi ASA, Miles B, Slawin KM (2005). Anomalies of the Wolffian Duct Derivatives Encountered at Radical Prostatectomy. Rev Urol.

[13] Sheih C-P, Hung C-S, Wei C-F, Lin C-Y (1990). Cystic dilatations within the pelvis in patients with ipsilateral renal agenesis or dysplasia. J Urol.

[14] Trigaux J-P, Van Beers B, Delchambre F (1991). Male genital tract malformations associated with ipsilateral renal agenesis: sonographic findings. J Clin Ultrasound.

[15] Walls WJ, Lin F (1975). Ultrasonic diagnosis of seminal vesicle cysts. Radiology.

[16] Shebel HM, Farg HM, Kolokythas O, El-Diasty T (2013). Cysts of the lower male genitourinary tract: embryologic and anatomic considerations and differential diagnosis. Radiographics.

[17] Ikari O, Castilho LN, Lucena R, D’Ancona CA, Netto NR (1999). Laparoscopic excision of seminal vesicle cysts. J Urol.

[18] Mege JL, Sabatier-Laval E, Mure PY, Vargas B, Dubois R, Takvorian P, Dodat H (1997). Malformations des organes génitaux masculins issus du canal de Wolff (épididyme, déférent, vésicule séminale, canal éjaculateur). Prog Urol.

[19] Cheng D, Amin P, Van Ha T (2012). Percutaneous sclerotherapy of cystic lesions. Semin Intervent Radiol.

